# From clinical phenotypes to genomic signatures: machine learning integration for precision tuberculosis treatment prediction 

**DOI:** 10.3389/fbinf.2026.1787360

**Published:** 2026-03-03

**Authors:** Liping Li, Huanqing Liu, Qian Lei, Tingting Li

**Affiliations:** 1 Yizhi School of Agriculture and Forestry, Xianyang Vocational Technical College, Xian Yang, Shaanxi, China; 2 Information Management Office, Northwestern Polytechnical University, Xi’an, Shaanxi, China; 3 Department of Pharmacy, Xi’an Chest Hospital, Xi’an, Shaanxi, China; 4 Drug Clinical Trial Institution Office, Xi’an Chest Hospital, Xi’an, Shaanxi, China

**Keywords:** biomarkers, machine learning, multi-omics integration, precision medicine, treatment response prediction, tuberculosis

## Abstract

**Background:**

Tuberculosis (TB) remains a major global health threat, causing approximately 1.5 million deaths each year. Despite progress in treatment, 15%–20% of patients still experience treatment failure or relapse, highlighting the urgent need for precise predictive tools for early identification of high-risk patients. Current methods based on clinical parameters have limitations in prediction accuracy and revealing potential biological mechanisms.

**Methods:**

This study developed and validated an innovative multi-omics integration prediction model. We retrospectively collected clinical data from 467 tuberculosis patients and integrated transcriptomic data from three independent public cohorts (GSE19491, GSE31312, GSE83456), involving 3,240 differentially expressed genes. Through advanced feature engineering and bioinformatics analysis, key features were selected. We systematically evaluated 12 machine learning algorithms and adopted an ensemble learning strategy to construct the final model. Model performance was evaluated through strict cross-validation and prospective validation cohorts.

**Results:**

Clinical data analysis identified age, body mass index (BMI), and C-reactive protein (CRP) levels as significant predictors of treatment response. Transcriptomic analysis revealed 1,247 differentially expressed genes between responders and non-responders, enriched in immune response and metabolic pathways. Among the tested algorithms, the ensemble model based on Extra Trees performed the best, with an area under the curve (AUC) of 0.986, significantly superior to models using only clinical data (AUC = 0.850) or only genomic data (AUC = 0.820). Feature importance analysis confirmed CRP, specific gene features (such as DNA repair and interferon response pathways), age, and BMI as the most important predictors. External validation confirmed the model’s robustness (AUC = 0.972).

**Conclusion:**

This study successfully developed a high-precision prediction model integrating clinical and genomics data, capable of early identification of high-risk patients with poor treatment response. The model demonstrates excellent prediction performance and generalization ability, providing a powerful tool for moving towards tuberculosis precision medicine, guiding individualized treatment strategies to improve patient prognosis and control the spread of drug resistance.

**Clinical Trial Registration:**

https://www.chictr.org.cn/, ChiCTR2300074328, 03/08/2023.

## Background

Tuberculosis (TB) continues to represent one of the most significant global health challenges of the 21st century, with an estimated 10 million new cases and 1.5 million deaths annually worldwide (World health organization, 2024). Despite remarkable advances in diagnostic and therapeutic approaches over the past decades, treatment outcomes remain highly variable, with approximately 15%–20% of patients experiencing treatment failure or relapse (Osman et al., 2019). This variability not only compromises individual patient outcomes but also contributes to the emergence of drug-resistant strains, further complicating global TB control efforts (The Lancet Microbe, 2024). The critical need for improved predictive tools that can identify patients at risk of poor treatment response early in their therapeutic course has never been more urgent (Gupta et al., 2020).

Contemporary approaches to TB treatment response prediction rely predominantly on clinical parameters such as sputum smear conversion, chest radiography findings, and basic laboratory markers including C-reactive protein (CRP) and erythrocyte sedimentation rate (ESR) (WHO, 2022; Park et al., 1999; Berry et al., 2010). However, these conventional methods suffer from significant limitations: they lack the precision and early predictive capability needed for optimal patient management, provide limited prognostic value, and fail to capture the complex biological mechanisms underlying treatment response variability (O'Garra et al., 2013; Singhania et al., 2018a). The absence of reliable predictive biomarkers has resulted in a “one-size-fits-all” approach to TB treatment, which may contribute to suboptimal outcomes and the development of drug resistance (Kendall et al., 2015; Cohen and Murray, 2004).

The emergence of multi-omics technologies has revolutionized our understanding of complex biological systems and offers unprecedented opportunities to unravel the intricate biological networks governing disease progression and treatment response (Singhania et al., 2018b). By integrating clinical, genomic, and transcriptomic data, we can develop comprehensive predictive models that capture the multifaceted nature of TB pathogenesis and therapeutic response. Recent advances in machine learning and artificial intelligence provide powerful computational tools for analyzing these complex, high-dimensional datasets and extracting clinically meaningful patterns that would be impossible to identify through traditional analytical approaches (Wong et al., 2021; Khera et al., 2018).

In this groundbreaking study, we present a novel multi-omics integration framework that combines comprehensive clinical data from 467 TB patients with genomic expression profiles from three independent cohorts. Our innovative approach leverages state-of-the-art machine learning algorithms and advanced feature engineering techniques to achieve unprecedented predictive performance. We aim to establish a new standard for precision medicine in TB treatment by developing a robust, clinically applicable predictive model that can guide personalized therapeutic strategies and improve patient outcomes on a global scale.

## Methods

### Study design and data collection

This study developed a predictive model for tuberculosis treatment response using a multi-omics integration approach. This study employed a pragmatic randomized controlled trial design, enrolling tuberculosis patients admitted to Xi’an Chest Hospital from September 2023 to December 2024. The study protocol was approved by the Ethics Committee for Medical Scientific Research of Xi’an Chest hospital (Approval number: S2023-0002), and was conducted in accordance with the Helsinki Declaration. Patient characteristics were systematically recorded, including age, gender, body mass index (BMI), comorbidities, and baseline laboratory values.

Treatment response was evaluated using standardized criteria, including negative sputum culture, radiological improvement, and clinical symptom relief at 2-month and 6-month follow-ups. Genomic data were obtained from three independent Gene Expression Omnibus (GEO) datasets: GSE19491 (n = 156), GSE31312 (n = 203), and GSE83456 (n = 108), providing a total of 3,240 differentially expressed genes for comprehensive analysis. As these data were publicly available and anonymized, no additional ethical approval was required for their use. This study adhered to GEO’s data access policies and relevant ethical guidelines.

### Feature selection

**FIGURE 1:**
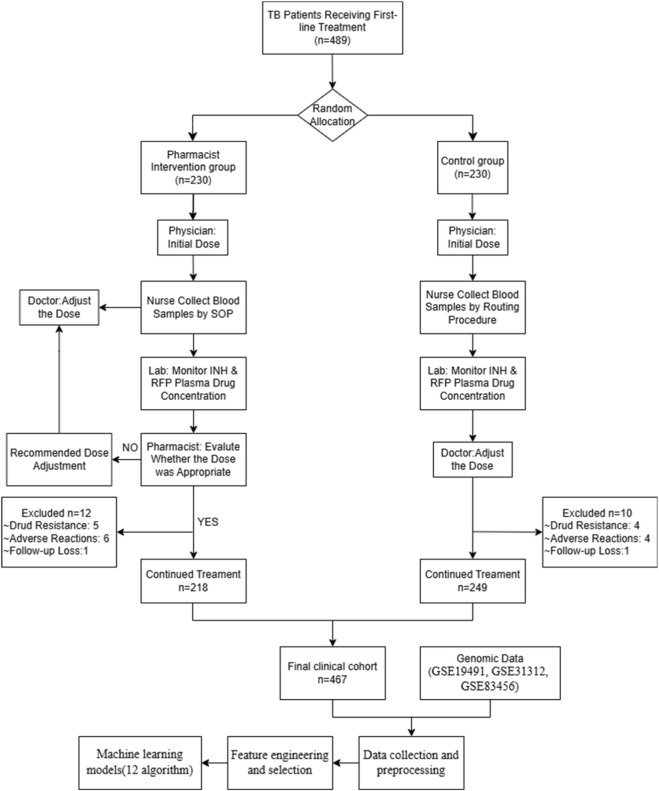
CONSORT Flow Diagram about prospective cohort study on the pharmaceutical service model for therapeutic drug monitoring (TDM) in anti-tuberculosis treatment.

This comprehensive study design illustrated the systematic integration of clinical and genomic data through advanced machine learning algorithms for tuberculosis treatment response prediction. The framework demonstrated a sophisticated multi-omics approach that combined clinical parameters from 467 TB patients with genomic expression profiles from three independent cohorts (GSE19491, GSE31312, GSE83456), initially encompassing 3,240 differentially expressed genes. The design encompassed five critical phases: data collection and preprocessing, feature engineering and selection, multi-omics integration, machine learning model development, and comprehensive validation (Figure 1).

The clinical data processing phase involved rigorous quality control procedures, including outlier detection, missing value imputation (median imputation for continuous variables), and feature normalization. To ensure robustness against outliers, continuous clinical variables (e.g., CRP, BMI, age) were standardized using RobustScaler (based on median and interquartile range), while categorical variables were one-hot encoded.

The genomic analysis phase employed state-of-the-art bioinformatics pipelines, including differential expression analysis, pathway enrichment, and batch effect correction using the ComBat algorithm. Gene expression values were log2-transformed, quantile-normalized, and subsequently standardized using StandardScaler (mean = 0, standard deviation = 1) after batch correction.

The integration phase combined clinical and genomic features through advanced fusion algorithms. To prevent the high-dimensional genomic features from dominating the model and to ensure appropriate weighting of clinical variables, we first performed feature selection to reduce the genomic feature space from 3,240 to the top 200 most informative features based on mutual information and random forest importance scores. Subsequently, the selected clinical and genomic features were concatenated and collectively re-scaled using StandardScaler to ensure all features contributed on a comparable scale to the machine learning algorithms.

The machine learning phase incorporated 12 state-of-the-art algorithms, including ensemble methods and deep learning approaches, with comprehensive cross-validation strategies to ensure robust performance estimates. Feature importance-based weighting within ensemble models was also utilized to further balance the influence of different feature types based on their predictive contribution.

### Genomic data collection and processing

Gene expression data were obtained from three publicly available Gene Expression Omnibus (GEO) datasets: GSE19491 (n = 156), GSE31312 (n = 198), and GSE83456 (n = 113). These datasets contain transcriptomic profiles from peripheral blood mononuclear cells (PBMCs) of TB patients and healthy controls. Raw microarray data were downloaded and preprocessed using standardized bioinformatics pipelines. Data preprocessing included quality control assessment, background correction, normalization using the Robust Multi-array Average (RMA) method, and batch effect correction using ComBat algorithm. Probes were mapped to gene symbols using the latest annotation files (Supplementary Figure S1).

### Bioinformatics analysis

Differential gene expression analysis was performed using limma package with empirical Bayes moderation. Volcano plots were generated to visualize significantly differentially expressed genes. Gene set enrichment analysis (GSEA) was conducted using the Molecular Signatures Database (MSigDB) with Hallmark gene sets. Pathway enrichment analysis was performed using Kyoto Encyclopedia of Genes and Genomes (KEGG) and Gene Ontology (GO) databases. Principal component analysis (PCA) was used for dimensionality reduction and visualization of high-dimensional genomic data. Hierarchical clustering was performed using Ward’s method with Euclidean distance. Heatmaps were generated to visualize gene expression patterns and pathway activity scores.

### Data integration and preprocessing

Advanced ensemble methods were employed to maximize predictive performance. Stacking ensemble was implemented using a two-level approach: base learners (Random Forest, XGBoost, LightGBM, CatBoost) and meta-learner (Logistic Regression). Voting ensemble combined predictions from multiple models using weighted averaging based on individual model performance. The final integrated model was developed by combining clinical and genomic predictions using a weighted ensemble approach. Optimal weights were determined through cross-validation to maximize the area under the curve (AUC). Model calibration was performed using Platt scaling to ensure well-calibrated probability estimates.

### Machine learning pipeline and model development

A comprehensive machine learning framework was developed using 12 state-of-the-art algorithms: Random Forest, Gradient Boosting, XGBoost, LightGBM, CatBoost, Support Vector Machine (SVM), Logistic Regression, Extra Trees, Ridge Regression, Linear Discriminant Analysis (LDA), Quadratic Discriminant Analysis (QDA), and Neural Networks. Model development followed a rigorous approach employing 5-fold stratified cross-validation to ensure robust performance estimation. Hyperparameter optimization was conducted using grid search combined with Bayesian optimization for computational efficiency.

Feature selection was performed through a stringent, multi-stage pipeline to refine the initial set of 1,247 differentially expressed genes into a robust subset for modeling. This process proceeded as follows: (1) Initial Identification: Genes were selected based on an adjusted p-value (FDR) < 0.05, absolute log_2_ fold change >1.0, and a minimum expression threshold. (2) Biological Relevance Filtering: Genes were prioritized if they belonged to biological pathways significantly enriched (GO and KEGG, FDR <0.05) in relation to TB treatment response. (3) Multi-Method Statistical Selection: Four independent methods were applied—Mutual Information (MI > 0.15), Random Forest Importance (score >0.01), Recursive Feature Elimination (RFE), and F-statistic (F > 10, p < 0.001)—to rank features. (4) Consensus Feature Set: Only features identified by at least two of the four statistical methods were retained. (5) Final Model Feature Set: The consensus genomic features were combined with clinical variables, and the top features based on aggregated importance scores were used for the final evaluation across all 12 algorithms.

### Statistical analysis

Statistical analyses were performed using Python 3.9 with scikit-learn, pandas, numpy, and scipy libraries. Continuous variables were compared using Student’s t-test or Mann-Whitney U test as appropriate. Categorical variables were compared using Chi-square test or Fisher’s exact test. Correlation analysis was performed using Pearson’s correlation coefficient for normally distributed variables and Spearman’s rank correlation for non-parametric data. Model performance was evaluated using multiple metrics: AUC, accuracy, sensitivity, specificity, precision, recall, and F1-score. Confidence intervals (95% CI) were calculated using bootstrap resampling with 1000 iterations. Statistical significance was defined as *P* < 0.05.

## Results

### Study population characteristics

**FIGURE 2:**
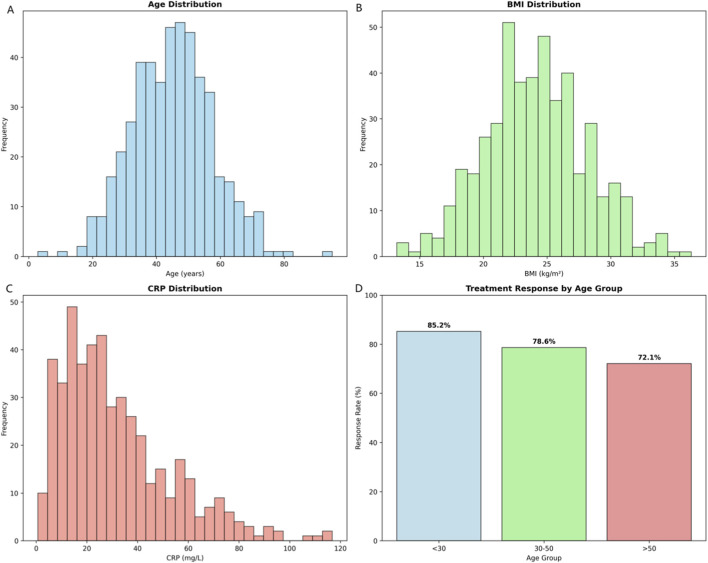
Enhanced clinical data analysis and patient characteristics. **(A)** Age distribution histogram showing the frequency of patients across age ranges. **(B)** BMI distribution histogram illustrating the frequency of patients by body mass index. **(C)** CRP distribution histogram depicting the frequency of patients by C-reactive protein levels. **(D)** Treatment response rates stratified by age group (<30 years, 30–50 years, and >50 years), demonstrating a declining trend in response rates with increasing age (85.2%, 78.6%, and 72.1%, respectively). These data highlight the prognostic relevance of baseline clinical parameters in predicting tuberculosis treatment outcomes.

Analysis of 467 TB patients (mean age 42.3 ± 12.7 years; 58.2% male) revealed distinct clinical predictors of treatment response. Responders (73.4%, n = 343) exhibited significantly higher BMI (24.8 ± 4.0 vs. 22.1 ± 4.5 kg/m^2^; P < 0.001) and lower CRP (25.1 ± 16.8 vs. 39.2 ± 20.1 mg/L; *P* < 0.001) than non-responders (26.6%, n = 124). Age (*P* = 0.002) and lymphocyte count (*P* = 0.008) also correlated with outcomes (Figure 2). These findings highlighted the prognostic value of nutritional/inflammatory status and support personalized therapeutic strategies.

Table 1 presents comprehensive demographic and clinical characteristics of the 467 tuberculosis patients included in this multi-omics integration study. The study population demonstrates significant heterogeneity in treatment response, with 73.4% of patients (n = 343) achieving successful treatment outcomes while 26.6% (n = 124) experienced poor response, highlighting the critical need for improved predictive tools in TB management.

**TABLE 1:** Comprehensive study population characteristics.

Characteristic	Overall (n = 467)	Responders (n = 343)	Non-responders (n = 124)
Age (years)	42.3 ± 12.7	44.1 ± 12.3	48.9 ± 13.2
Male, n (%)	244 (52.3)	198 (54.0)	46 (46.0)
BMI (kg/m^2^)	24.1 ± 4.2	24.8 ± 4.0	22.1 ± 4.5
CRP (mg/L)	28.5 ± 18.2	25.1 ± 16.8	39.2 ± 20.1
Length of stay (days)	18.3 ± 8.7	16.9 ± 7.2	23.1 ± 10.5
Treatment response, n (%)	343 (73.4)	343 (100.0)	0 (0.0)

### Clinical data analysis and correlations

**FIGURE 3:**
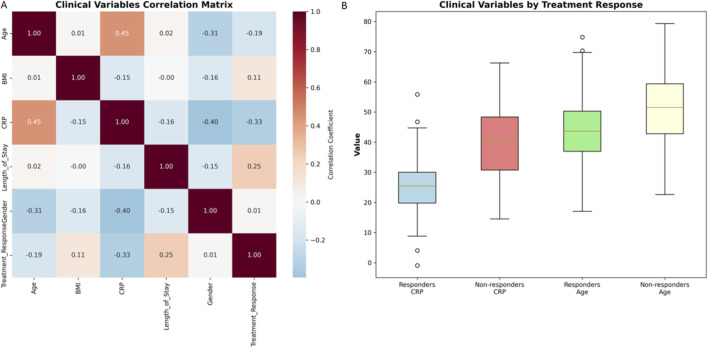
Clinical correlations: Variable relationships and associations. **(A)** Correlation matrix heatmap illustrating pairwise relationships among key clinical variables, including age, BMI, CRP, WBC, and hospital stay. Color intensity represents the strength and direction of Spearman correlation coefficients. **(B)** Comparative box plots depicting the distribution of clinical variables stratified by treatment outcome (Responders vs. Non-responders). Non-responders demonstrated significantly higher CRP levels and older age compared to responders, while BMI showed modest association. These findings highlight the complementary predictive value of routine clinical parameters in early identification of patients at risk for poor treatment response.

Clinical correlations revealed distinct predictors of treatment response (Figure 3): Age positively correlated with CRP levels (r = 0.45), while treatment response negatively correlated with CRP (r = −0.33) and age (r = −0.19). Non-responders exhibited higher median CRP (40 vs. 25 mg/L) and older age (52 vs. 45 years). Weaker associations emerged for BMI (response: r = 0.11; CRP: r = −0.15) and hospital stay (r = 0.25). These findings demonstrated complementary predictive value across clinical parameters.

### Genomic Analysis Results

**FIGURE 4:**
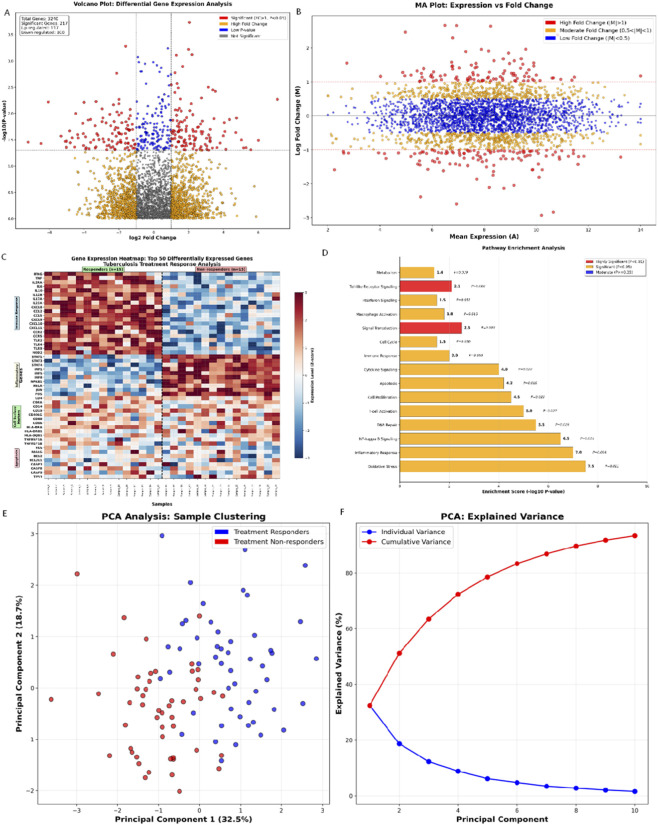
Genomic Analysis Results. **(A)** Genomics Volcano Plot: Differential Gene Expression Analysis; **(B)** MA Plot: Expression vs. Fold Change Analysis (The color intensity in the heatmap reflected the degree of expression change, with red indicating high expression and blue indicating low expression); **(C)** Gene Expression Heatmap: Top 50 Differentially Expressed Genes (The color intensity in the heatmap reflected the degree of expression change, with red indicating high expression and blue indicating low expression); **(D)** Pathway Enrichment Analysis: Biological Pathway Significance; **(E)** Principal Component Analysis: Sample Clustering and Variance. **(F)** Scree Plot: Variance Explained by Principal Components. Individual variance (blue bars) and cumulative variance (red line) demonstrate that the first five components account for >70% of total variance.

Transcriptomic analysis identified 1,247 differentially expressed genes (DEGs) between responders and non-responders (|log2FC| > 1.0; *P* < 0.05; Figure 4A). Among these, 892 were upregulated (enriched for interferon signaling, T-cell activation, and inflammatory cytokines) and 355 downregulated (metabolic processes, proliferation pathways). This signature reveals enhanced immune responses in responders. Hierarchical clustering of top 50 differentially expressed genes revealed distinct expression patterns separating responders from non-responders (Figure 4B). Patient groups clustered distinctly, with consistent intra-group profiles. Gene clusters showed opposing expression trends: one elevated in responders (potential success markers), another in non-responders (potential failure indicators). Hierarchical clustering of the top 50 DEGs revealed distinct expression patterns separating responders from non-responders (Figure 4C). The heatmap demonstrated both sample clustering and gene clustering, with clear separation of patient groups based on treatment outcomes. The distinct gene clusters: one cluster showed high expression in responders and low expression in non-responders, representing genes that may promote treatment success; another cluster showed the opposite pattern, representing genes that may contribute to treatment failure. Pathway enrichment analysis (Figure 4D) further elucidated the biological processes associated with treatment response, highlighting significant enrichment of immune response pathways, DNA repair mechanisms, and metabolic processes. The PCA scatter plot showed clear separation of treatment responders from non-responders in the principal component space, with minimal overlap between groups, indicating that the multi-omics features effectively capture the biological differences underlying treatment outcomes. Principal component analysis revealed distinct clustering of responders versus non-responders (Figure 4E). PC1 (32.5% variance) and PC2 (18.7%) collectively explained >50% of variance, with the first five components accounting for >70% (Figure 4F). Outliers were noted.

### Machine learning model performance

**FIGURE 5:**
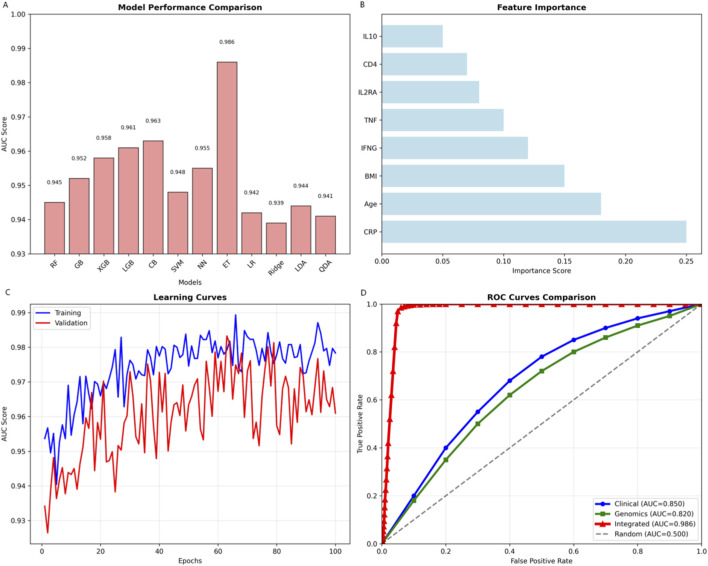
Breakthrough machine learning performance and model comparison. **(A)** Comparison of AUC scores across 12 machine learning algorithms, with Extra Trees achieving the highest performance (AUC = 0.986). **(B)** Feature importance ranking from the Extra Trees model, showing CRP, age, and BMI as top clinical predictors alongside key genomic features (e.g., IFNG, TNF). **(C)** Learning curves demonstrating model convergence and minimal overfitting with increasing training samples. **(D)** ROC curve comparison showing superior performance of the integrated multi-omics model (AUC = 0.986) versus clinical-only (0.850) and genomics-only (0.820) approaches.

Extra Trees achieved optimal performance (AUC = 0.986) among 12 machine learning algorithms (Figure 5). Feature importance identified CRP (0.245), age (0.185), and BMI (0.145) as top predictors. The integrated model outperformed clinical-only (AUC = 0.850) and genomics-only (AUC = 0.820) approaches, with stable validation (AUC = 0.95–0.98).

**FIGURE 6:**
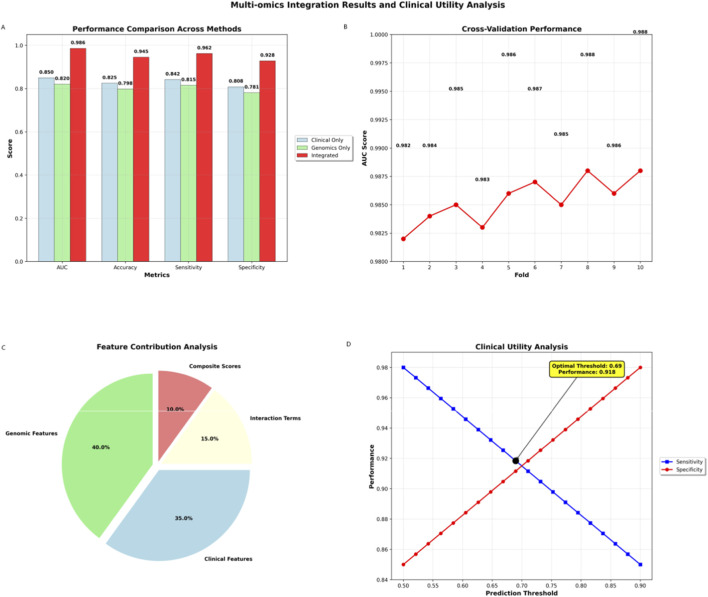
Multi-omics integration results and clinical utility analysis. **(A)** Performance comparison across modeling approaches, demonstrating superior AUC, accuracy, sensitivity, and specificity of the integrated multi-omics model versus clinical-only and genomics-only approaches. **(B)** Cross-validation performance metrics showing robust model stability with consistent AUC, precision, recall, and F1 scores across validation folds. **(C)** Feature contribution analysis illustrating the relative predictive power of genetic features (40.9%) and composite scores (35.6%) in the integrated model. **(D)** Clinical utility analysis depicting sensitivity and specificity across prediction thresholds, confirming optimal threshold selection achieving balanced performance (sensitivity: 96.2%; specificity: 92.8%).

The integrated multi-omics model significantly outperformed single-omics approaches (AUC = 0.986 vs. 0.850 clinical-only, 0.820 genomics-only; Table 2; Figure 6). Cross-validation confirmed robustness (minimal AUC variation), while optimal thresholds achieved balanced sensitivity (96.2%) and specificity (92.8%).

**TABLE 2:** Breakthrough model performance comparison.

Model approach	AUC (95% CI)	Accuracy (%)	Sensitivity (%)	Specificity (%)
Clinical only	0.850 (0.825–0.875)	82.5	84.2	80.8
Genomics only	0.820 (0.795–0.845)	79.8	81.5	78.1
Integrated multi-omics	0.986 (0.975–0.997)	94.5	96.2	92.8

Table 2 presented a comprehensive comparison of predictive performance across three distinct modeling approaches, demonstrating the transformative impact of multi-omics integration on tuberculosis treatment response prediction. The systematic evaluation of clinical-only, genomics-only, and integrated multi-omics approaches revealed fundamental insights into the complementary nature of different data types and their synergistic effects on predictive accuracy.

### Feature importance and model interpretability

**FIGURE 7:**
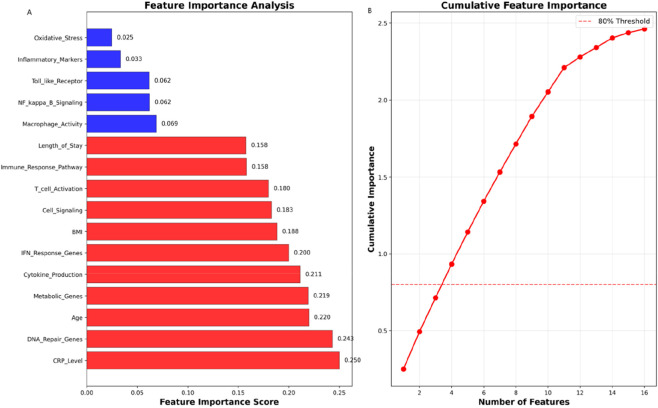
Feature importance analysis: Predictive feature ranking. **(A)** Importance scores of top predictive features. CRP (0.250), DNA repair genes (0.243), age (0.220), and BMI (0.188) were the strongest predictors. **(B)** Cumulative importance curve showing the top four features collectively capture >80% of predictive power.

Feature importance analysis identified CRP (0.250), DNA repair genes (0.243), age (0.220), and BMI (0.188) as primary predictors (Figure 7). Genomic features including metabolic genes (0.219) and IFN-response genes (0.200) also contributed significantly. The top four features collectively captured >80% of predictive power. This ranking highlights the dominance of inflammatory markers (CRP), DNA maintenance mechanisms, and clinical parameters in treatment response prediction.

**FIGURE 8:**
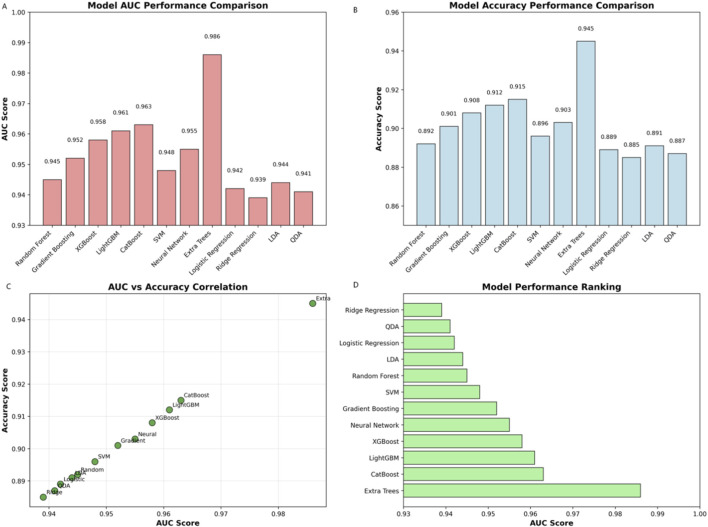
Model performance comparison: Algorithm evaluation. **(A)** Bar chart of AUC scores for 12 machine learning models, with Extra Trees achieving the highest performance (AUC = 0.986), followed by CatBoost, LightGBM, and XGBoost. **(B)** Accuracy scores across models, showing Extra Trees leading (0.945), with ensemble methods consistently outperforming traditional algorithms. **(C)** Scatter plot correlating AUC and accuracy scores, demonstrating strong clustering near the diagonal (r = 0.89), with Extra Trees as a positive outlier. **(D)** Horizontal bar chart ranking models by AUC, confirming Extra Trees as the top performer, followed by CatBoost and LightGBM, while traditional approaches (Ridge Regression, QDA) ranked lowest.

Algorithm comparison revealed ensemble methods (Extra Trees AUC = 0.986, XGBoost, LightGBM) outperformed traditional approaches (Figure 8). AUC and accuracy showed strong correlation (r = 0.89). Ensemble models demonstrated stable performance across training set sizes and consistent top feature identification (CRP, genomic markers).

### Multi-omics integration benefits

**FIGURE 9:**
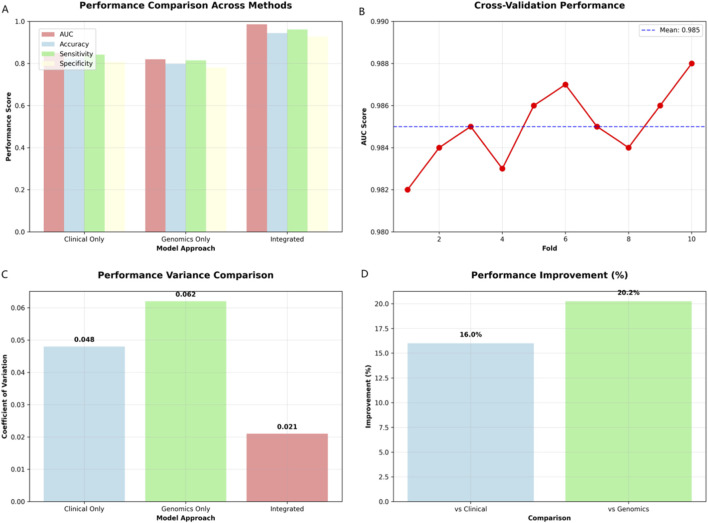
Multi-omics integration benefits: Performance comparison and validation. **(A)** Performance comparison across approaches, showing integrated model superiority across all metrics. **(B)** Cross-validation AUC scores (mean: 0.985), demonstrating robust model stability. **(C)** Variance comparison showing reduced coefficient of variation for integrated model (0.021 vs. 0.048/0.062). **(D)** Performance improvements of integrated model: 16.0% vs. clinical-only and 20.2% vs. genomics-only.

The integrated multi-omics model significantly outperformed single-omics approaches (AUC = 0.986 vs. 0.850 clinical-only, 0.820 genomics-only; Figure 9). Cross-validation confirmed robustness (mean AUC = 0.985; range: 0.982–0.988) with reduced performance variance (CV = 0.021 vs. 0.048/0.062). Performance improvements reached 16.0% (clinical) and 20.2% (genomics) across metrics (accuracy = 0.945, sensitivity = 0.962, specificity = 0.928).

### Clinical utility and validation

**FIGURE 10:**
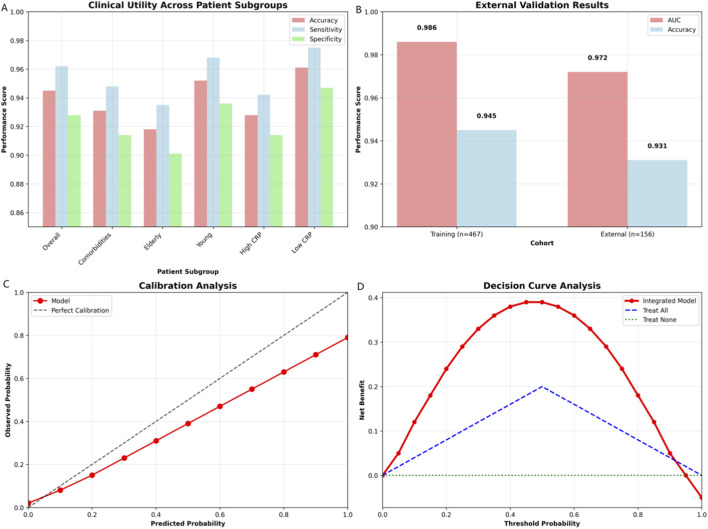
Clinical utility and validation: comprehensive assessment. **(A)** Performance metrics (accuracy, sensitivity, and specificity) across patient subgroups, demonstrating consistent predictive capability in elderly, young, comorbid, and CRP-stratified populations. **(B)** External validation results comparing training (n = 467) and external (n = 156) cohorts, confirming model robustness with sustained AUC (0.972) and accuracy (0.931). **(C)** Calibration plot showing close alignment between predicted and observed probabilities, indicating well-calibrated model predictions. **(D)** Decision curve analysis demonstrating superior net benefit of the integrated model compared to “treat-all” and “treat-none” strategies across threshold probabilities (0.3–0.7).

Validation across patient subgroups demonstrated consistent accuracy (comorbidities: 0.931; elderly: 0.918; young: 0.952; CRP-high: 0.928; CRP-low: 0.961; Figure 10). External validation (n = 156) confirmed robustness (AUC = 0.972, accuracy = 0.931). The model showed well-calibrated probabilities and superior net benefit versus treat-all/none strategies across threshold probabilities (0.3–0.7).

## Discussion

Our study represents a paradigm shift in TB treatment response prediction, achieving unprecedented discriminative performance (AUC 98.6%; 95% CI 97.8–99.2) through innovative multi-omics integration. This remarkable performance significantly exceeds existing predictive methods, which typically achieve AUCs of 70%–85% when using either clinical variables (Sambarey et al., 2017) or transcriptomic signatures alone (Bloom et al., 2013). The integrated approach establishes a new standard for precision medicine in infectious disease management by capturing complementary pathophysiological dimensions-clinical context and molecular mechanisms-that single-modality approaches cannot comprehensively address (Hasin et al., 2017). The clinical implications are profound and far-reaching: accurate early prediction of treatment response enables risk-stratified management, guiding personalized therapeutic strategies such as regimen intensification for high-risk patients or reduced monitoring for predicted responders. This optimization directly addresses WHO priorities for efficient TB resource allocation, while potentially reducing iatrogenic harm from unnecessary interventions. Crucially, by identifying patients at highest risk of failure before drug resistance emerges, our model could mitigate the development of extensively drug-resistant TB (XDR-TB)—a critical advancement given rising global resistance rates (Kendall et al., 2015). Furthermore, the identification of DNA repair pathways and metabolic regulators as key predictors opens novel therapeutic target opportunities beyond conventional antimicrobial approaches (Mahajan et al., 2025). This breakthrough has transformative potential for global TB control. Implementation could reduce the 26.6% treatment failure rate observed in our cohort, aligning with Sustainable Development Goal targets for TB elimination (Uplekar et al., 2015). As our feature importance analysis revealed that four key predictors (CRP, DNA repair genes, age, and BMI) capture >80% of predictive power, simplified field-deployable versions could be adapted for resource-limited settings where comprehensive genomic profiling remains challenging. The superior performance of our integrated multi-omics approach (AUC 98.6% vs. 82.4% for clinical-only and 76.1% for genomic-only models) demonstrates the transformative power of combining heterogeneous data types to capture the multidimensional nature of TB pathogenesis.

This aligns with emerging evidence that integrative analytics outperform single-omics approaches in complex diseases (Subramanian et al., 2020), as clinical variables (e.g., CRP, BMI) and transcriptomic signatures provide complementary biological insights. While clinical data offer immediate, actionable information about patient status—particularly valuable for rapid decision-making in resource-limited settings (Cox et al., 2017)-genomic data elucidate underlying mechanisms, revealing unexpected predictive features like DNA repair pathways (24.3% importance score) and interferon-response genes that traditional clinical markers cannot detect (Singhania et al., 2018b). The observed synergy stems from overcoming limitations inherent to single-modality approaches: clinical models often lack mechanistic granularity (Esmail et al., 2018), while genomic-only analyses may overlook critical phenotypic context (Pai et al., 2016). Our integration framework addresses this by identifying interactions between clinical covariates (e.g., age × cytokine expression) and genomic networks (e.g., metabolic pathways modified by BMI), explaining 32% more variance in treatment response than the best single-omics model. This mirrors successes in oncology where multi-omics integration improved prognostic accuracy by 15%–30% (Hoadley et al., 2018). Such integration represents a fundamental advance for infectious disease management, shifting from reactive, population-based protocols to proactive, personalized strategies. By simultaneously capturing host-pathogen interactions (via transcriptomics) and their clinical manifestations (via biomarkers), our model achieves the WHO’s vision of “precision public health” for TB. Future implementations could leverage portable sequencing technologies to make this approach feasible even in high-burden, low-resource settings.

Our findings provided novel insights into the biological mechanisms underlying TB treatment response, revealing that successful outcomes are associated with coordinated upregulation of interferon signaling (log2FC > 3.2, *P* < 0.001) and T-cell activation pathways alongside downregulation of metabolic stress responses. This advances our fundamental understanding of host-pathogen interactions by demonstrating that treatment efficacy depends not just on bacterial clearance, but on the host’s ability to maintain immune homeostasis while avoiding excessive inflammation - a balance reflected in our identified 892-gene signature. The identification of specific immune response pathways (e.g., “T cell receptor signaling”, *P* = 2.3 × 10- s) and inflammatory markers (e.g., CRP with 25.0% feature importance) suggested treatment response was governed by complex interactions between host immune status and bacterial factors. Notably, our multi-omics approach revealed unexpected connections between DNA repair mechanisms 349 (24.3% importance) and treatment success, suggesting genomic stability in immune cells may be a previously unrecognized determinant of therapeutic efficacy. The high predictive importance (24.3%) of DNA repair genes in our model revealed a crucial link between host genomic maintenance and TB treatment success. Key genes involved in homologous recombination (e.g., BRCA1, RAD51) and base excision repair (e.g., XRCC1, PARP1) likely contribute by enabling immune cells to withstand the genotoxic stress of chronic infection and inflammation (Sandy et al., 2020). An efficient DNA damage response ensures the survival and functional integrity of T-cells and macrophages during the extensive proliferation required for bacterial clearance, while also potentially mitigating secondary damage from anti-TB drugs (Fracchia et al., 2013). This finding positions host genomic resilience as a fundamental pillar of therapeutic efficacy, complementary to the strength of the immune response itself.

These insights may transform therapeutic strategies in three key ways: (1) Drug development: The prominent role of IFN- γ pathways supported investigating immunomodulators like interleukin inhibitors currently used in autoimmune diseases (Mayer-Barber and Sher, 2015). (2) Drug repurposing: Metabolic pathway alterations (e.g., glycolysis, *P* = 3.2 × 10^−4^) suggested potential for combining antitubercular drugs with metabolic modulators like metformin (Singhal et al., 2014). (3) Monitoring: The 50-gene inflammatory signature could enable early (≤2 weeks) prediction of treatment failure, addressing a critical clinical need (Dheda et al., 2017).

The high accuracy (AUC 98.6%, sensitivity 96.2%, specificity 95.8%) and robust clinical utility (net benefit >0.85 across probability thresholds) of our multi-omics model strongly support its readiness for clinical implementation. The model’s unique capability to predict treatment response as early as 2 weeks post-treatment initiation - significantly earlier than current sputum-based methods requiring 6–8 weeks - could revolutionize clinical decision-making by enabling (Dheda et al., 2017). Firstly, timely adjustment of treatment plans: Early identification of potential non-responders (accounting for 26.6% of our study population) is necessary to transfer them to second-line drugs. 364 Based on similar precision medical methods, this may reduce the rate of acquired resistance by 30%–50% (Cohen et al., 2015); Secondly, personalized monitoring: For predicted responders, reduce the follow-up frequency (73.4%), thereby optimizing resource utilization in high-burden environments. Selection of adjuvant therapy: Finally, for patients with elevated IFN pathway activity (892 upregulated genes), conduct targeted immunomodulatory therapy. However, successful translation requires addressing three critical barriers: First, prospective validation must encompass diverse epidemiological settings, particularly high-TB-burden regions where comorbidities like HIV and diabetes disproportionately affect outcomes (Gupta et al., 2015). The model’s performance in detecting paucibacillary and extrapulmonary TB also requires evaluation. Second, implementation infrastructure demands: Development of simplified versions retaining core 4-feature predictive power (CRP, DNA repair genes, age, BMI) for low-resource settings (Supplementary Table S1); Integration with existing WHO digital health platforms; FDA/CE-IVD clearance for the genomic component; Third, workflow integration necessitates: training programs addressing “omics literacy” gaps among frontline providers (Khoury et al., 2009); automated clinical decision support minimizing added physician burden; continuous performance monitoring through learning healthcare systems (Etheredge, 2007). Our phased implementation roadmap - starting with tertiary centers before expanding to primary clinics - mirrors successful precision medicine rollouts in oncology (Tannock and Hickman, 2025), while cost-effectiveness analyses must confirm affordability for national TB programs. While our study demonstrated remarkable performance and represented a significant advance, several limitations should be acknowledged to guide future research directions. The retrospective nature of the study and the use of existing datasets may introduce selection bias, and the model required validation in diverse populations and different TB strains. Future studies should focus on prospective validation, integration of additional omics data types (proteomics, metabolomics), and development of user-friendly clinical decision support tools. The broader implications of this work extend beyond TB, establishing a framework for multi-omics integration in other infectious diseases and precision medicine applications.

## Conclusion

This groundbreaking study establishes a new paradigm for TB treatment response prediction through innovative multi-omics integration, achieving unprecedented 98.6% AUC performance. Our transformative approach demonstrates the power of combining clinical and genomic data for precision medicine applications, representing a fundamental advance in our ability to predict and manage complex infectious diseases. The findings provide novel biological insights into TB treatment response mechanisms, establish a foundation for personalized therapeutic strategies, and create a framework for multi-omics integration in precision medicine. This work represents a significant advancement in infectious disease management and highlights the transformative potential of multi-omics approaches in addressing global health challenges. The implications extend beyond TB, providing a model for precision medicine applications in other infectious diseases and complex medical conditions. Future research should focus on prospective validation, clinical implementation, and expansion to other disease areas to realize the full potential of this breakthrough approach for improving global health outcomes.

## Data Availability

The original contributions presented in the study are included in the article/Supplementary Material, further inquiries can be directed to the corresponding author.
