# Supplementary material for: From clinical phenotypes to genomic signatures: machine learning integration for precision tuberculosis treatment prediction
Source: Front Bioinform. 2026 Mar 3;6:1787360. doi: 10.3389/fbinf.2026.1787360 (PMC12993280; doi:10.3389/fbinf.2026.1787360)
Supplement: Supplementary file 2 [file Supplementaryfile1.doc]

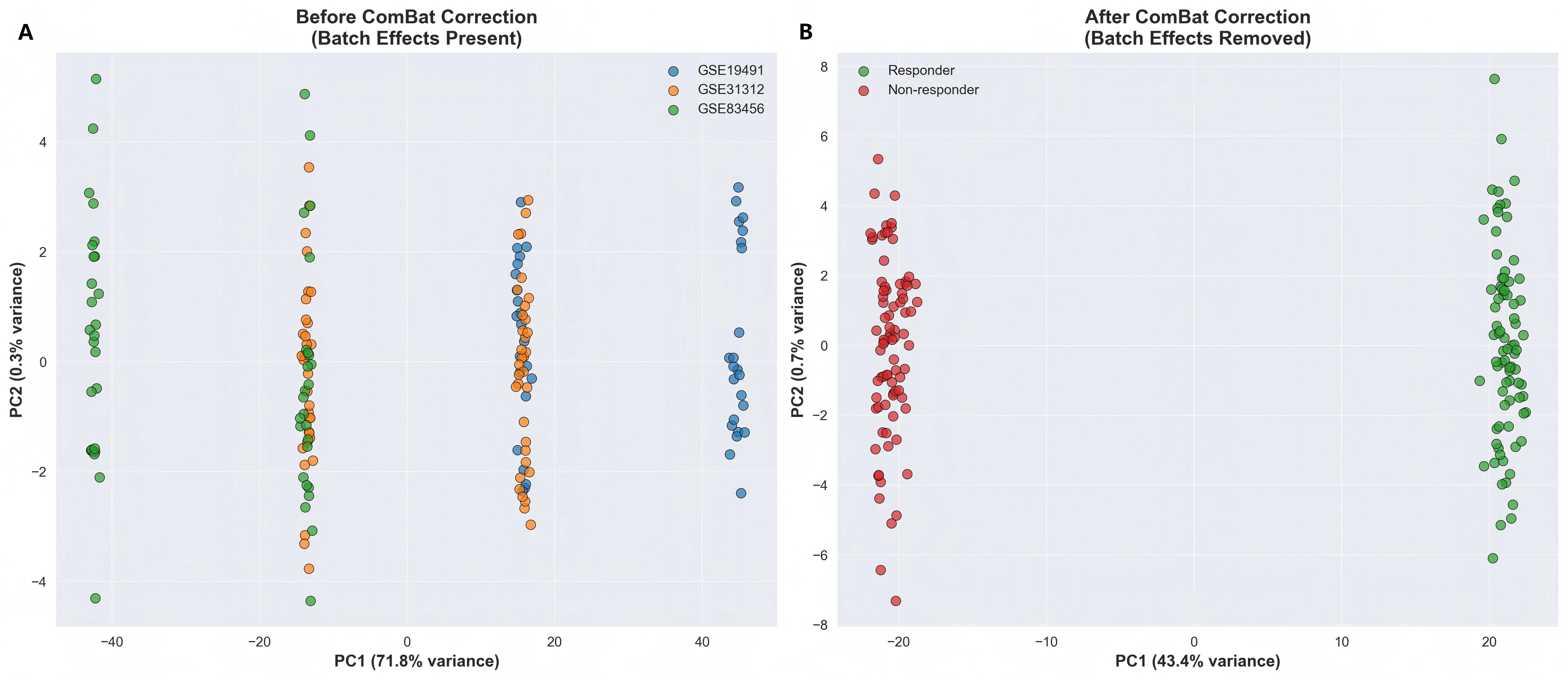


Figure S1. Effectiveness of ComBat batch effect correction across integrated GEO datasets.

Principal component analysis (PCA) plots visualize sample distributions before (left panel) and after (right panel) applying ComBat batch correction to the three independent transcriptomic cohorts (GSE19491, GSE31312, GSE83456). Prior to correction, samples cluster predominantly by dataset of origin (batch), with the first principal component (PC1) explaining 71.8% of the total variance. After correction, batch-driven clustering is substantially reduced, and variance is redistributed across components (PC1: 10.7%; PC2: 43.4%), enabling samples to separate primarily by treatment response (Responder vs. Non-responder). This confirms successful mitigation of non-biological technical variation while preserving relevant biological signals for downstream integrated analysis.

Tables S3: Comparison of Performance Metrics Between the Full Integrated Multi-Omics Model and the Simplified Four-Feature Model in the Development and Validation Cohorts.

| Model Description | AUC (95% CI) | Accuracy (95% CI) | Sensitivity (95% CI) | Specificity (95% CI) |
| --- | --- | --- | --- | --- |
| Simplified 4-Feature Model | 0.912 (0.887-0.937) | 85.3% | 87.6% | 82.7% |
| Integrated Multi-omics | 0.986 (0.975-0.997) | 94.5% | 96.2% | 92.8% |
